# The Value of the Naples Prognostic Score and the Systemic Immune-Inflammation Index in Predicting Ischemia on Myocardial Perfusion Scintigraphy

**DOI:** 10.3390/diagnostics15111372

**Published:** 2025-05-29

**Authors:** Hakan Süygün, Damla Yalçınkaya Öner, Ugur Nadir Karakulak

**Affiliations:** 1 Department of Cardiology, Faculty of Medicine, Karamanoğlu Mehmetbey University, 70110 Karaman, Turkey; 2 Karaman Training and Research Hospital, 70200 Karaman, Turkey; damlaykaya@gmail.com; 3 Department of Cardiology, Faculty of Medicine, Hacettepe University, 06230 Ankara, Turkey; ukarakulak@gmail.com

**Keywords:** stable angina pectoris, myocardial perfusion scintigraphy, myocardial ischemia, Naples prognostic score, systemic immune-inflammation index

## Abstract

**Objectives:** Early identification of myocardial ischemia is critical for the management of patients with stable angina pectoris (SAP). The Naples Prognostic Score (NPS) and the Systemic Immune-Inflammation (SII) index are emerging biomarkers that may improve risk stratification prior to myocardial perfusion scintigraphy (MPS) **Methods:** We retrospectively analyzed 615 patients with SAP who underwent MPS to assess the predictive value of the NPS and SII index for myocardial ischemia. Clinical, laboratory, and imaging data were collected. The associations between the NPS, SII, and ischemia detected on MPS were evaluated through univariate and multivariate logistic regression analyses. **Results:** A higher NPS was strongly associated with the presence of myocardial ischemia (*p* < 0.001). Male sex, elevated SII, and increased C-reactive protein (CRP) and neutrophile-to-lymphocyte ratio (NLR) values were also significantly related to ischemia. In multivariate analysis, NPS (*p* < 0.001), SII (*p* = 0.023), CRP (0.005), and NLR (0.037) values remained independent predictors of ischemia. Albumin levels were significant in univariate analysis but lost independent significance after adjustment. The incorporation of the NPS and SII index provided additional value in identifying patients at high risk of ischemia. **Conclusions:** The NPS and the SII index are inexpensive, very simple, non-invasive, and valuable markers of myocardial ischemia in patients with SAP. Their integration into clinical practice may enhance risk stratification and optimize diagnostic pathways, minimizing unnecessary invasive procedures.

## 1. Introduction

Cardiovascular diseases remain the leading cause of mortality worldwide, with coronary artery disease (CAD) accounting for a significant proportion of morbidity and healthcare burden [1]. Atherosclerosis, the primary pathological mechanism underlying CAD, is now recognized as a chronic inflammatory process [2,3]. The inflammatory process not only contributes to plaque formation but also destabilizes existing plaques, leading to acute coronary events [4,5]. It has been established that immune system and inflammatory cells including neutrophils, lymphocytes, monocytes, and platelets play an essential role in the occurrence of coronary atherosclerosis [6,7].

In addition to inflammation, nutritional status has been increasingly recognized as a contributing factor in atherosclerosis progression. Low serum albumin levels have been associated with increased inflammation, endothelial dysfunction, and worse cardiovascular outcomes [8,9]. The relationship between inflammation, nutritional status, and CAD progression highlights the importance of discovering novel biomarkers.

The Naples Prognostic Score (NPS) has emerged as a novel composite biomarker, integrating systemic inflammation and nutritional status. It incorporates serum albumin levels, total cholesterol (TC), neutrophil-to-lymphocyte ratios (NLRs), and lymphocyte-to-monocyte ratios (LMRs) to provide a comprehensive assessment of patient prognosis. NPS was first described in colorectal cancer by Galizia et al. It has subsequently been extensively studied in oncology [10,11,12]. However, recent evidence suggests that it may also be useful in cardiovascular diseases [13,14,15,16,17].

Similarly, the systemic immune-inflammation index (SII), initially developed for use in oncology, is a novel biomarker that integrates platelet, neutrophil, and lymphocyte counts, reflecting both systemic inflammation and immune response. SII has shown promise in predicting clinical outcomes in various cardiovascular conditions, including acute coronary syndromes and chronic heart failure [18,19,20,21,22]. Elevated SII levels have been associated with increased disease severity and worse clinical outcomes in CAD patients, suggesting its potential role as a simple yet effective risk stratification tool.

Angina pectoris, precipitated by ischemia, is the most prevalent manifestation of atherosclerotic CAD. The main diagnostic technique used to identify CAD in patients with stable angina pectoris (SAP) is non-invasive imaging scans. In light of recent guidelines, myocardial perfusion scintigraphy (MPS) is a widely used noninvasive imaging technique that allows the assessment of myocardial ischemia and perfusion defects, guiding clinical decision making in patients with suspected or known CAD [23,24]. MPS using single-photon emission computed tomography (SPECT) is a popular diagnostic method for identifying myocardial functional ischemia in patients with suspected CAD.

The correlation of inflammatory and nutritional markers with functional ischemia, documented by MPS, remains an area requiring further investigation. Given their well-established correlation with CAD, identifying accessible and cost-effective biomarkers associated with functional ischemia on MPS could significantly improve risk stratification. The present study aims to evaluate the predictive value of SII and the NPS in identifying myocardial ischemia in patients with SAP, thereby providing insight into their potential role integrating inflammatory and nutritional markers with functional imaging in CAD assessment and diagnosis. To the best of our knowledge, this is the first study to evaluate both the NPS and the SII index as predictors of functional myocardial ischemia assessed by MPS in patients with SAP.

## 2. Methods

### 2.1. Patient Population and Study Design

This is a single-centere, retrospective study of patients diagnosed with stable angina pectoris. A total of 1186 patients who were diagnosed with SAP and underwent myocardial perfusion imaging (MPI) according to the established guidelines at Karaman Training and Research Hospital between May 2024 and January 2025 were included in the study.

**Figure 1:**
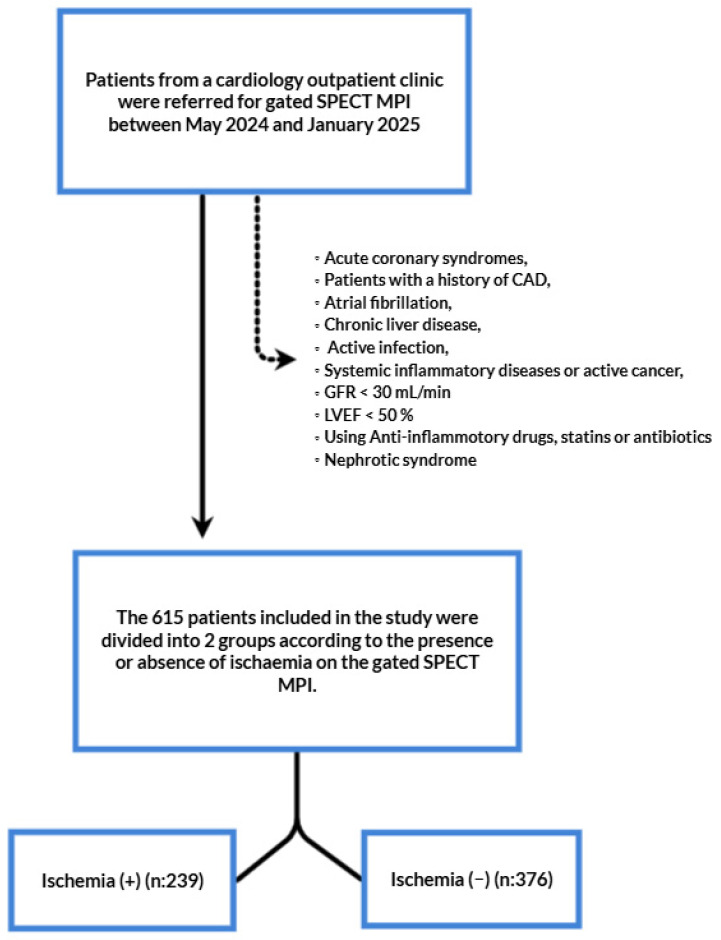
Flow chart of the study. SPECT: single-photon emission computed tomography; MPI: myocardial perfusion imaging; CAD: coronary artery disease; GFR: glomerular filtration rate; LVEF: left ventricular ejection function.

Inclusion criteria were defined as follows: patients over 18 years of age who had undergone MPI with a diagnosis of SAP. Patients were categorized into two groups: no ischemia and ischemia. We used the traditional clinical classification of SAP that met the following criteria: constricting discomfort in the front of the chest or in the neck, jaw, shoulder, or arm, precipitated by physical exertion and relieved by rest or nitrates within five minutes, and the continuation of these symptoms for more than two months. According to the same guidelines, MPI was performed on those who had a moderate-to-high (15–85%) clinical likelihood of obstructive CAD [25]. Exclusion criteria were defined as follows: acute coronary syndromes, patients with a history of CAD (including percutaneous or surgical revascularization), diseases that can affect serum albumin, total lymphocyte, monocyte, and neutrophil count, and TC levels including metabolic syndrome, nephrotic syndrome, severe renal impairment (defined as a creatinine clearance less than 30 mL/L and/or the need for renal replacement therapy), chronic liver disease, active infection, systemic inflammatory diseases and active malignancy, current or previous use of lipid lowering medications, current use of anti-inflammatory drugs, and patients with insufficient data for calculation of Naples score and SII. Following the application of exclusion criteria, a total of 615 patients were examined. Figure 1 shows the flowchart of the study.

Our study complied with the Declaration of Helsinki and was approved by the local Ethical Committee of the Karamanoglu Mehmetbey University, Karaman, Turkey. Patients or the public were not involved in the design, conduct, reporting or dissemination plans of our study.

### 2.2. Data Collection and Analysis

Information on demographic characteristics, previously diagnosed diseases like hypertension (HT), diabetes mellitus (DM), hyperlipidemia (HL), and chronic kidney disease, history of smoking, and previous medications was obtained from medical records. DM was defined as a fasting glucose > 126 mg/dL, HbA1c > 6.5% or a history of antidiabetic medications [26]. HT was defined as a systolic blood pressure ≥ 140 mmHg and diastolic blood pressure ≥ 90 mmHg and/or a history of antihypertension treatment at enrollment [27]. HL was defined as a total cholesterol level > 240 mg/dL [28].

### 2.3. Laboratory Measurements

Blood samples taken from patients on the day of their outpatient clinic admission were recorded from the database. Routine blood tests included complete blood counts, serum biochemical tests (renal and liver functions, C-reactive protein (CRP, mg/dL), high-density lipoprotein (HDL, mg/dL), low-density lipoprotein (LDL, mg/dL), triglycerides (mg/dL), and TC (mg/dL). An automated hematology analyzer (Mindray BC-6000, Mindray Bio-Medical Electronics Co., Shenzhen, China) was used to measure hematological indices. In addition, creatinine, serum electrolytes, serum cholesterol levels, and detailed liver function tests were measured with a Beckman Coulter AU5800 modular analyzer (Beckman Coulter Inc., Brea, CA, USA).

We divide the total neutrophil count by the lymphocyte count, and the total lymphocyte count by the monocyte count to calculate the NLR and LMR, respectively. The NPS was calculated based on the original definition proposed by Galizia et al. [10], using four components: NLR, LMR, TC level, and serum albumin level. Each of these parameters is assigned a score of either 0 (NLR ≤ 2.96, LMR > 4.44, TC > 180 mg/dL, serum albumin ≥ 4 mg/dL) or 1 (NLR > 2.96, LMR ≤ 4.44, TC ≤ 180 mg/dL serum albumin < 4 mg/dL) and the scores are summed. Patients were then evaluated as low NPS group (0–1–2) and high NPS group (3–4) according to NPS. The SII index was calculated using the following formula from the blood count: platelet count × NLR.

### 2.4. Myocardial Perfusion Imaging

All patients underwent myocardial perfusion scintigraphy (MPS) using a standardized two-day stress/rest protocol with Technetium-99m methoxy isobutyl isonitrile (Tc-99m MIBI). Patients were instructed to fast for a minimum of six hours prior to imaging and to avoid caffeine-containing products for a period of 24 h before pharmacologic stress testing.

The stress protocol involved treadmill exercise using the modified Bruce protocol. At the point of peak exercise (target heart rate = [220 − age] × 0.85), 20 mCi of Tc-99m MIBI was administered intravenously, after which exercise continued for a further minute. For patients unable to exercise, adenosine was infused intravenously at a rate of 140 µg/kg/min for six minutes, with 20 mCi of Tc-99m MIBI injected at the third minute (peak hyperemia).

Stress imaging was initiated 30–45 min following injection. Patients exhibiting perfusion defects on stress imaging underwent rest imaging with an additional 20 mCi of Tc-99m MIBI, acquired 30–45 min later. SPECT images were obtained using a dual-head gamma camera (Siemens Symbia, Germany) with SMARTZOOM™ collimators over a 180° arc (45° right anterior oblique to 45° left posterior oblique), using a 64 × 64 matrix, 3° intervals, and 60 projections per head. The analysis of perfusion defects was conducted semi-quantitatively using the Total Stress Score (TSS), Total Rest Score (TRS), and Total Difference Score (TDS), with the grading of ischemia as normal (TSS < 4), mild (TSS 4–8), moderate (TSS 9–13), or severe (TSS > 13). Two experienced nuclear medicine physicians independently reviewed the images, with any discrepancies resolved by consensus.

### 2.5. Statistical Analysis

Data analysis was performed using IBM SPSS Statistics version 25.0 software (IBM Corporation, Armonk, NY, USA). The Kolmogorov–Smirnov test was used to investigate whether the normal distribution assumption was met. Categorical data were expressed as numbers (*n*) and percentages (%), while quantitative data were given as mean ± SD and median (25th–75th) percentiles. While the mean differences between the groups were compared using the Student’s *t*-test, the Mann–Whitney U test was used to compare data that did not show a normal distribution. Qualitative data were analyzed by Pearson’s χ^2^ test. Receiver operating characteristic (ROC) curve analyses were performed to determine potential cut-off values for the NLR, LMR, and SII as predictors of ischemia development. Where the area under the curve (AUC) was statistically significant, the optimal cut-off point was identified using Youden’s index. The sensitivity, specificity, positive predictive value (PPV), negative predictive value (NPV), and accuracy were also calculated. To identify independent predictors of ischemia, multiple logistic regression models were constructed. Any variable with a *p* < 0.15 in univariate analysis was considered for inclusion in the multivariate model. For each independent variable, odds ratios (ORs) with 95% confidence intervals (CIs) and Wald statistics were reported. A *p*-value < 0.05 was considered as statistically significant.

## 3. Results

The study included a total of 615 patients divided into two groups: no ischemia (*n* = 376) and ischemia (*n* = 239). Baseline characteristics are displayed in Table 1. The mean age was 61.6 ± 9.5 and 366 (59.5%) were male. Compared to the non-ischemic group, the ischemic group had a statistically significant lower proportion of women and a higher proportion of men (*p* < 0.001). Body mass indexes (BMIs), comorbidities like HT, DM, and HL, and smoking history were statistically similar between two groups (*p* > 0.05).

**Figure 2:**
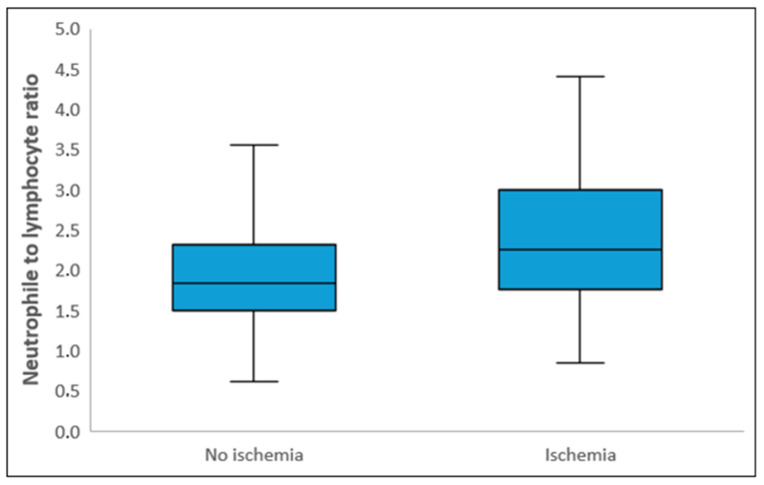
Box-plot graph of neutrophile-to-lymphocyte ratio (NLR) in patients with and without ischemia. The lines in the center of each box represent the median NLR levels, while the lower and upper edges of the boxes correspond to the 25th percentile and 75th percentile values of the NLR levels, respectively. The vertical sections extending from the lower and upper edges of the boxes and continuing upwards and downwards represent the minimum and maximum values, respectively.

**Figure 3:**
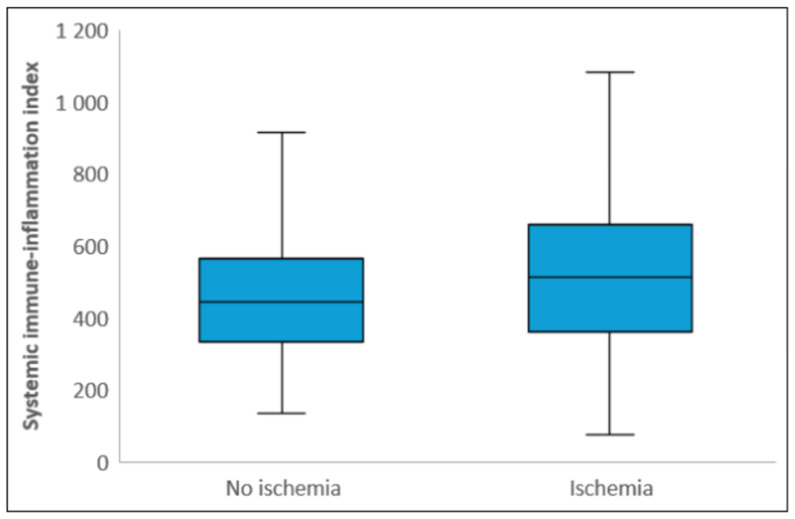
Box plot of Systemic Immune-Inflammation index (SII) in patients with and without ischemia. The lines in the center of each box represent the median SII levels, while the lower and upper edges of the boxes correspond to the 25th percentile and 75th percentile values of the SII levels, respectively. The vertical sections extending from the lower and upper edges of the boxes and continuing upwards and downwards represent the minimum and maximum values, respectively.

**Figure 4:**
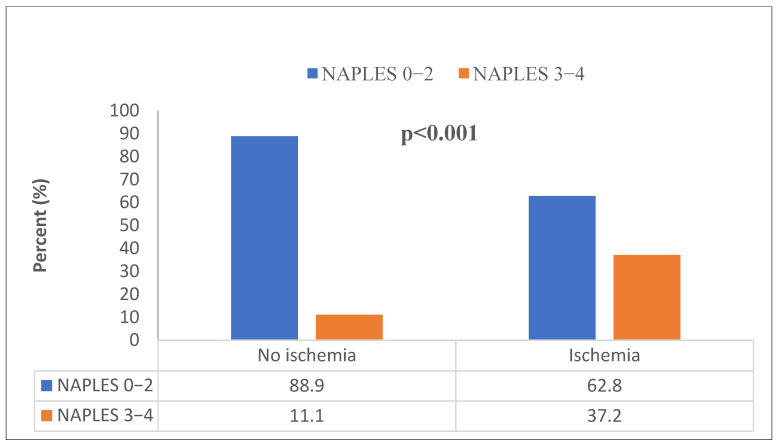
The distribution low and high NPS in ischemic and non-ischemic groups. (Pearson’s χ^2^ test.).

Laboratory parameters showed that white blood cell count, neutrophil count, CRP, and total cholesterol levels were significantly higher in the ischemic group, while albumin and PLT levels were significantly lower compared to the non-ischemic group (*p* < 0.05). NLR and SII levels were also significantly higher in the ischemic group (*p* < 0.001) (Figure 2 and Figure 3). The median NPS was 1 (0–2) in the non-ischemic group and 2 (2–3) in the ischemic group (*p* < 0.001). The distribution of those with low and high NPSs in the ischemic and non-ischemic groups is shown in Figure 4. While 334 (88.9%) of the patients in the non-ischemic group had low NPSs, 89 (37.2%) of the patients in the ischemic group had high NPSs (*p* < 0.001).

**Figure 5:**
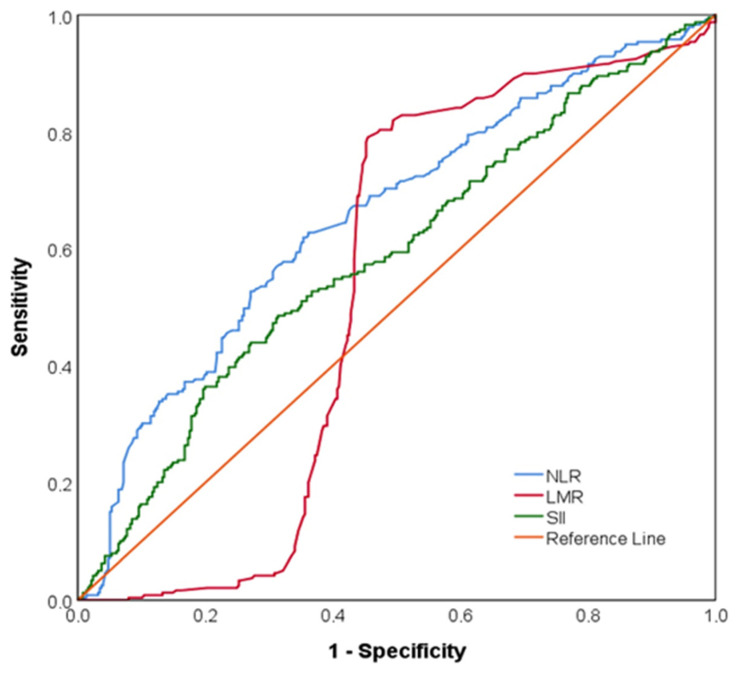
ROC curves of neutrophile-to-lymphocyte ratio (NLR), lymphocyte-to-monocyte ratio (LMR) and Systemic Immune-Inflammation index (SII).

Figure 5 shows the ROC curves of NLR, LMR, and SII levels to predict ischemia. NLR levels above 2.04 predicted ischemia with a sensitivity of 62.8% and specificity of 63.8% (AUC = 0.656 [95% CI: 0.611–0.700], *p* < 0.001). SII levels above 528.27 predicted ischemia with an area under the ROC curve = 0.588 [95% CI: 0.542–0.634] (*p* < 0.001). The area under the ROC curve of LMR measurements was statistically insignificant in distinguishing the two groups (AUC = 0.534, [95% CI: 0.487–0.581], *p* = 0.159) (Supplementary Materials Table S1).

Multivariable logistic regression analysis showed a high NPS (OR = 4.427 [2.642–7.923], 95% CI; *p* < 0.001), male sex (OR = 6.792 [4.168–11.068], 95% CI; *p* = 0.004), higher CRP levels (OR = 1.181 [1.046–1.333], 95% CI; *p* = 0.007), and NLR above 2.04 (OR = 1.580 [1.028–2.429], 95% CI; *p* < 0.037) were independent predictors of ischemia (Table 2). Due to multicollinearity between NLR and SII, these variables were not included simultaneously in the regression model. In Model 2, SII was incorporated into the analysis instead of NLR, which was excluded from this model to avoid redundancy with the first model. After multivariate adjustment, a high NPS (OR = 4.945 [2.913–8.767], 95% CI; *p* < 0.001), male sex (OR = 7.250 [4.430–11.865], 95% CI; *p* < 0.001), higher CRP levels (OR = 1.191 [1.053–1.348], 95% CI; *p* = 0.005), and SII above 528.27 (OR = 1.676 [1.072–2.621], 95% CI; *p* < 0.023) were independent predictors of ischemia (Table 3).

## 4. Discussion

To the best our knowledge, this study is the first to evaluate the utility of the NPS and SII as predictors of functional myocardial ischemia detected by MPS. Our findings demonstrate that NPS, a composite score reflecting both inflammatory and nutritional status, serves as a robust independent predictor of ischemia, with a high NPS (scores 3–4) associated with a 4.4- to 4.9-fold increase in the likelihood of ischemia. Male sex and higher CRP, NLR, and SII values also emerged as significant predictors, reinforcing the interplay between systemic inflammation and ischemic burden in stable CAD. The findings highlight several key aspects regarding the interplay between inflammation, nutritional status, and the risk of myocardial ischemia.

Atherosclerosis is a common disease with significant clinical consequences, including CAD. Identifying myocardial ischemia in patients with SAP remains crucial for optimal clinical management and prognosis. Early recognition of ischemia allows for timely initiation of medical therapy, risk factor modification, and, when necessary, revascularization procedures. Patients with CAD typically develop myocardial ischemia. The genesis of atherosclerosis and myocardial ischemia is complex and involves several biological mechanisms, including biomolecular and inflammatory processes [29,30]. During the early phases of myocardial ischemia, inflammatory responses occur in myocardial tissue. CRP, albumin, and NLR have been proposed as potential biomarkers for inflammation, particularly in the context of acute coronary syndromes [31,32]. Numerous studies have identified NLR as a significant predictor of acute and stable CAD [4,33,34,35]. Previous studies have highlighted the importance of the CRP/albumin ratio (CAR) as a sensitive and accessible inflammatory index in cardiovascular disease. In two separate studies, elevated CAR was found to be an independent predictor of both the severity of myocardial ischemia on MPS and the extent of CAD evaluated by angiography [36,37]. To investigate the relationship between myocardial perfusion and the NLR, platelet-to-lymphocyte ratio, platelet distribution width, and RDW, Ozdemir et al. [38] studied 262 patients with abnormal and normal MPS. Those diagnosed with myocardial ischemia or infarction had significantly higher neutrophil counts and NLRs. Similarly, in our study, CRP, WBC, neutrophils and NLRs among inflammatory markers were higher in the ischemia group.

In a recent study where the NPS was only assessed in 110 patients with MPS and 37 patients in the ischemic group, albumin and the NPS were found to be predictors [39]. Our univariate analyses showed that low levels of albumin were associated with the presence of ischemia in MPS, as has been found in similar studies evaluating CAR and NPS [37,39,40]. This is consistent with the literature suggesting hypoalbuminemia reflects a chronic inflammatory state and malnutrition, both of which are known to contribute to atherogenesis and myocardial vulnerability [8,41]. However, albumin alone did not retain significance in multivariable models, which underscores the added prognostic value of integrated scores like NPS over individual laboratory parameters. These findings reinforce the pathophysiological link between systemic inflammation, nutritional depletion, and ischemic burden. These findings are also consistent with literature highlighting the prognostic significance of SII and the NPS in cardiovascular disease. For instance, Lai et al. demonstrated that the NPS was an effective predictor of mortality in patients with cardiovascular diseases [42]. Similarly, a meta-analysis by Zhao et al. confirmed that elevated SII levels are significantly associated with major adverse cardiovascular events in patients with CAD [43]. Our results extend this perspective by demonstrating that the NPS—which integrates albumin, total cholesterol, NLR, and LMR values—provides an even stronger association with ischemia. This supports the hypothesis that composite biomarkers better capture the multifactorial nature of CAD progression than individual laboratory parameters.

This is the first study to investigate the SII index for myocardial functional ischemia on MPS in stable angina patients. The statistically significant relationship between the higher SII index and myocardial ischemia was another notable finding of our analysis. The SII index integrates platelet counts with NLRs (NLR × platelet count), suggesting a complex interaction between the immune response and hemostatic balance in the setting of CAD. High SII levels have been shown to be significantly associated with poorer clinical outcomes in several studies of cardiovascular disease and have proven useful as a simple risk stratification tool in clinical practice [17,19,20,21,22,44]. It has been discovered that platelets contribute to the development of CAD [45]. Plaque content contains chemokines such platelet factor 4–5, and platelet activation has also been shown to actively contribute to plaque formation [46,47]. In our study, high NLR values in the ischemic group were statistically significant in multivariate analysis, in agreement with the literature. However, contrary to expectations, platelet count was lower in the ischemic group in univariate analysis and not statistically significant in multivariate analysis. This finding may be explained by the established inverse relationship between albumin and platelet levels, with increased albumin levels having been shown to decrease platelet reactivity and prevent thrombosis [6]. Although platelet count alone was lower in the ischemic group, SII, which combines platelet count with the NLR, remained independently associated with ischemia. This apparent contradiction may be explained by the disproportionately elevated NLR in the ischemic group, which was sufficient to drive SII upward despite relatively lower platelet counts. In other words, the strong contribution of neutrophilia and lymphopenia outweighed the modest decline in platelet values when calculating SII. This finding also highlights the advantage of composite indices like the SII index, which reflect dynamic interactions among multiple inflammatory parameters rather than relying on a single marker. Additionally, previous studies have shown that platelets may become functionally more reactive even when absolute counts are lower in chronic inflammatory states, further supporting the relevance of SII in cardiovascular risk assessment [45,46]. The predictive capacity of SII, though statistically significant, was more modest (AUC 0.588) compared to the NPS. This might reflect differences in the inflammatory mechanisms driving stable versus acute CAD. While SII has demonstrated prognostic value in acute coronary syndromes, its role in chronic ischemia appears less pronounced in this study. The lymphocyte-to-monocyte ratio (LMR) did not reach statistical significance, suggesting that neutrophil-predominant inflammation may be more relevant to ischemia detection in stable CAD. These findings underscore the need for context-specific biomarker selection based on disease acuity and phenotype. Our results extend prior work on inflammatory indices such as the CAR, NLR and SII, which have shown promise in risk stratification but lack the nutritional dimension incorporated in the NPS [35,37,40,44]. Prior studies have individually validated the use of SII and the NPS in various cardiovascular contexts. For instance, Yang et al. [19] demonstrated the prognostic value of SII in patients with CAD, while Dziedzic et al. [22] linked SII with both disease severity and systemic inflammation. In terms of CAR, Sabanoglu et al. [37] and Efe et al. [40] showed its correlation with ischemia severity and extent of coronary involvement. However, none of these studies evaluated the NPS and SII’s predictive capacity specifically for functional ischemia as assessed by MPS, highlighting the novel contribution of our work.

From a clinical perspective, the accessibility of the NPS and SII makes them practical tools for risk stratification. These scores offer several practical advantages in routine care. As both the NPS and SII are derived from simple, inexpensive, and widely available laboratory parameters, they can be integrated into standard outpatient evaluations without additional cost or delay. When used early in the diagnostic pathway, particularly in patients with SAP, these indices may help clinicians identify those who are less likely to have myocardial ischemia and could be safely monitored without immediate imaging. This may ultimately reduce unnecessary referrals for MPS or coronary angiography. Despite the retrospective design and single-center setting, our findings suggest that these indices have the potential to be incorporated into clinical algorithms to prioritize testing and personalize care. Incorporating these indices into the assessment of patients with SAP may enhance the identification of high-risk individuals who may benefit from intensified medical therapy or earlier referral for advanced imaging. For instance, patients with high NPSs may be prioritized for closer follow-up or a targeted nutrition and early intervention approach.

Several limitations warrant consideration. First, because of the retrospective design, we cannot determine cause-and-effect relationships, and it may overlook other factors that could affect the results, like differences in diet or unreported health issues. Second, the single-center cohort limits generalizability, and validation in broader populations is essential. Third, the inherent multicollinearity between the NLR and SII required us to create separate models, showing that these two indices reflect similar inflammatory processes. Future research should explore the longitudinal relationship between the NPS, SII, and hard clinical endpoints, including mortality and revascularization outcomes. Further studies are also required to investigate how malnutrition and inflammation interact to drive ischemia, which could reveal new treatment targets.

## 5. Conclusions

In conclusion, this study identifies the NPS and SII as important novel biomarkers for myocardial functional ischemia in patients with stable CAD. They integrate systemic inflammation and nutritional status into the assessment of myocardial ischemia. By incorporating these biomarkers into clinical practice, we can improve risk assessment and tailor treatment strategies, aiming to enhance outcomes for patients with SAP. It is crucial for future studies to confirm these findings in larger and diverse patient groups. Additionally, further research should investigate how interventions that target inflammation and nutrition can benefit high-risk patients, thereby improving our understanding of these biomarkers and their clinical significance.

## Figures and Tables

**Table 1:** Demographic, clinical characteristics and laboratory measurements of the patients according to the groups with and without ischemia.

	Overall (*n* = 615)	No Ischemia (*n* = 376)	Ischemia (*n* = 239)	*p*-Value
Age (years)	61.6 ± 9.5	61.1 ± 9.8	62.3 ± 9.0	0.130 ^A^
Gender				<0.001 ^B^
Female	249 (40.5%)	210 (55.9%)	39 (16.3%)	
Male	366 (59.5%)	166 (44.1%)	200 (83.7%)	
BMI (kg/m^2^)	25.5 ± 2.2	25.4 ± 2.2	25.6 ± 2.1	0.250 ^A^
HT	359 (58.4%)	217 (57.7%)	142 (59.4%)	0.677 ^B^
DM	153 (24.9%)	92 (24.5%)	61 (25.5%)	0.768 ^B^
HL	113 (18.4%)	67 (17.8%)	46 (19.2%)	0.656 ^B^
Smoking	141 (22.9%)	77 (20.5%)	64 (26.8%)	0.070 ^B^
LVEF	60.0 (55.0–62.0)	60.0 (56.0–62.0)	60.0 (55.0–62.0)	0.183 ^C^
Hemoglobin	14.2 ± 1.49	14.1 ± 1.47	14.3 ± 1.53	0.111 ^A^
Hematocrit	41.7 ± 4.1	41.5 ± 4.0	42.1 ± 4.2	0.051 ^A^
LDL	131.0 (113.0–149.0)	131.0 (107.2–148.0)	131.0 (118.0–151.0)	0.100 ^C^
HDL	48.0 (42.0–55.0)	48.0 (41.0–55.0)	48.0 (42.0–55.0)	0.852 ^C^
Triglyceride	132.0 (100.0–183.0)	130.0 (100.0–175.7)	133.0 (100.0–190.0)	0.550 ^C^
Total cholesterol	196.0 (171.0–244.0)	178.0 (165.2–250.0)	206.0 (184.0–236.0)	<0.001 ^C^
CRP	0.70 (0.50–1.50)	0.60 (0.50–1.20)	1.20 (0.60–2.10)	<0.001 ^C^
Creatinine	0.87 (0.72–1.00)	0.84 (0.70–1.00)	0.90 (0.75–1.01)	0.074 ^C^
Albumin	4.23 (3.93–4.43)	4.25 (4.12–4.40)	4.10 (3.68–4.50)	<0.001 ^C^
WBC	7.1 (6.0–8.5)	7.0 (6.0–8.4)	7.4 (6.3–8.8)	0.005 ^C^
PLT	230.0 (194.0–270.0)	235.5 (197.0–273.0)	220.0 (188.0–260.0)	0.021 ^C^
Neutrophile	4.6 (3.8–5.6)	4.2 (3.5–5.1)	5.1 (4.5–5.8)	<0.001 ^C^
Lymphocyte	2.3 (2.0–2.6)	2.3 (1.9–2.7)	2.3 (2.0–2.5)	0.508 ^C^
Monocyte	0.60 (0.50–0.80)	0.60 (0.50–0.90)	0.60 (0.60–0.80)	0.432 ^C^
NLR	2.00 (1.59–2.50)	1.84 (1.50–2.32)	2.26 (1.76–3.00)	<0.001 ^C^
LMR	3.8 (2.6–4.8)	4.5 (2.1–5.0)	3.7 (3.0–4.2)	0.161 ^C^
SII	459.5 (341.7–617.1)	445.3 (332.0–566.7)	513.0 (361.2–659.7)	<0.001 ^C^
Naples	2 (1–2)	1 (0–2)	2 (2–3)	<0.001 ^C^
Naples				<0.001 ^B^
Low (0–2)	484 (78.7%)	334 (88.9%)	150 (62.8%)	
High (3–4)	131 (21.3%)	42 (11.1%)	89 (37.2%)	

NLR: neutrophile-to-lymphocyte ratio, LMR: lymphocyte-to-monocyte ratio, SII: Systemic Immune-Inflammation index. Continuous variables are shown as mean ± SD or median (25th–75th) percentiles, where appropriate. ^A^ Student’s *t* test, ^B^ Pearson’s χ^2^ test, ^C^ Mann–Whitney U test.

**Table 2:** Examination of the multiple effects of all factors thought to be determinant in differentiating the groups with and without ischemia with multivariate logistic regression analysis results including NLR in the model equation.

	Odds Ratio	95% Confidence Interval	Wald	*p*-Value
Age	1.014	0.993–1.036	1.654	0.198
Male factor	6.792	4.168–11.068	59.131	<0.001
Total cholesterol	1.003	0.999–1.007	2.047	0.152
CRP	1.181	1.046–1.333	7.216	0.007
Albumin	0.682	0.394–1.182	1.860	0.173
WBC	1.061	0.980–1.148	2.123	0.145
Naples > 2	4.427	2.642–7.923	19.764	<0.001
NLR > 2.04	1.580	1.028–2.429	4.346	0.037

CRP, C-reactive protein; NLR, neutrophil-to-lymphocyte ratio; WBC, white blood cell count.

**Table 3:** Examination of the multiple effects of all factors thought to be determinant in differentiating the groups with and without ischemia with multivariate logistic regression analysis results including SII in the model equation.

	Odds Ratio	95% Confidence Interval	Wald	*p*-Value
Age	1.017	0.995–1.039	2.370	0.124
Male factor	7.250	4.430–11.865	62.110	<0.001
Total cholesterol	1.003	0.999–1.007	1.973	0.160
CRP	1.191	1.053–1.348	7.731	0.005
Albumin	0.693	0.401–1.199	1.718	0.190
WBC	1.051	0.968–1.140	1.416	0.234
Naples > 2	4.945	2.913–8.767	20.912	<0.001
SII > 528.27	1.676	1.072–2.621	5.136	0.023

CRP, C-reactive protein; SII, Systemic Immune-Inflammation index; WBC, white blood cell count.

## Data Availability

The data presented in this study are available upon request from the corresponding author. The data are not publicly available due to privacy and ethical restrictions.

## Supplementary Materials

The following supporting information can be downloaded at: https://www.mdpi.com/article/10.3390/diagnostics15111372/s1, Table S1: ROC analysis results of NLR, LMR and SII measurements in differentiating ischemia and non-ischemia groups.

## References

[1] Ralapanawa U., Sivakanesan R. (2021). Epidemiology and the magnitude of coronary artery disease and acute coronary syndrome: A narrative review. J. Epidemiol. Glob. Health.

[2] Libby P., Ridker P.M., Hansson G.K. (2009). Leducq Transatlantic Network on Atherothrombosis. Inflammation in atherosclerosis: From pathophysiology to practice. J. Am. Coll. Cardiol..

[3] Montecucco F., Liberale L., Bonaventura A., Vecchiè A., Dallegri F., Carbone F. (2017). The role of inflammation in cardiovascular outcome. Curr. Atheroscler. Rep..

[4] Ateş A.H., Aytemir K., Koçyiğit D., Yalcin M.U., Gürses K.M., Yorgun H., Canpolat U., Hazırolan T., Özer N. (2016). Association of neutrophil-to-lymphocyte ratio with the severity and morphology of coronary atherosclerotic plaques detected by multidetector computerized tomography. Acta Cardiol. Sin..

[5] Hansson G.K., Libby P., Tabas I. (2015). Inflammation and plaque vulnerability. J. Intern. Med..

[6] Davì G., Patrono C. (2007). Platelet activation and atherothrombosis. N. Engl. J. Med..

[7] Falk E. (2006). Pathogenesis of atherosclerosis. J. Am. Coll. Cardiol..

[8] Chien S.-C., Chen C.-Y., Leu H.-B., Su C.-H., Yin W.-H., Tseng W.-K., Wu Y.-W., Lin T.-H., Chang K.-C., Wang J.-H. (2017). Association of low serum albumin concentration and adverse cardiovascular events in stable coronary heart disease. Int. J. Cardiol..

[9] Suzuki S., Hashizume N., Kanzaki Y., Maruyama T., Kozuka A., Yahikozawa K. (2019). Prognostic significance of serum albumin in patients with stable coronary artery disease treated by percutaneous coronary intervention. PLoS ONE.

[10] Galizia G., Lieto E., Auricchio A., Cardella F., Mabilia A., Podzemny V., Castellano P., Orditura M., Napolitano V. (2017). Naples prognostic score, based on nutritional and inflammatory status, is an independent predictor of long-term outcome in patients undergoing surgery for colorectal cancer. Dis. Colon Rectum.

[11] Elia S., Patirelis A., Hardavella G., Santone A., Carlea F., Pompeo E. (2023). The Naples prognostic score is a useful tool to assess surgical treatment in non-small cell lung cancer. Diagnostics.

[12] Chen F., Xie C., Ren K., Xu X. (2023). Prognostic value of the Naples prognostic score in patients with gastrointestinal cancers: A meta-analysis. Nutr. Cancer.

[13] Çetin Z.G., Balun A., Çiçekçioğlu H., Demirtaş B., Yiğitbaşı M.M., Özbek K., Çetin M. (2023). A novel score to predict one-year mortality after transcatheter aortic valve replacement, Naples prognostic score. Medicina.

[14] Gitmez M., Ekingen E., Zaman S. (2025). Predictive Value of the Naples Prognostic Score for One-Year Mortality in NSTEMI Patients Undergoing Selective PCI. Diagnostics.

[15] Kılıç O., Suygun H., Mustu M., Karadeniz F.O., Ozer S.F., Senol H., Kaya D., Buber I., Karakurt A. (2023). Is the Naples prognostic score useful for predicting heart failure mortality. Kardiologiia.

[16] Erdogan A., Genc O., Ozkan E., Goksu M.M., Ibisoglu E., Bilen M.N., Guler A., Karagoz A. (2023). Impact of Naples prognostic score at admission on in-hospital and follow-up outcomes among patients with ST-segment elevation myocardial infarction. Angiology.

[17] Ozkan E., Erdogan A., Karagoz A., Tanboğa I.H. (2024). Comparison of systemic immune-inflammation index and Naples prognostic score for prediction coronary artery severity patients undergoing coronary computed tomographic angiography. Angiology.

[18] Hu B., Yang X.-R., Xu Y., Sun Y.-F., Sun C., Guo W., Zhang X., Wang W.-M., Qiu S.-J., Zhou J. (2014). Systemic immune-inflammation index predicts prognosis of patients after curative resection for hepatocellular carcinoma. Clin. Cancer Res..

[19] Hu B., Yang X.-R., Xu Y., Sun Y.-F., Sun C., Guo W., Zhang X., Wang W.-M., Qiu S.-J., Zhou J. (2020). Systemic immune-inflammation index (SII) predicted clinical outcome in patients with coronary artery disease. Eur. J. Clin. Investig..

[20] Dziedzic E.A., Gąsior J.S., Tuzimek A., Dąbrowski M., Jankowski P. (2022). The Association between Serum Vitamin D concentration and new inflammatory biomarkers—Systemic inflammatory index (SII) and systemic inflammatory response (SIRI)—In patients with ischemic heart disease. Nutrients.

[21] Özen Y., Erdöl A., Özbay M.B., Erdoğan M. (2023). The Prognostic Role of the Systemic Inflammatory Index (SII) in Heart Failure Patients: Systemic Inflammatory Index and Heart Failure. Int. J. Curr. Med. Biol. Sci..

[22] Dziedzic E.A., Gąsior J.S., Tuzimek A., Paleczny J., Junka A., Dąbrowski M., Jankowski P. (2022). Investigation of the associations of novel inflammatory biomarkers—Systemic inflammatory index (SII) and systemic inflammatory response index (SIRI)—With the severity of coronary artery disease and acute coronary syndrome occurrence. Int. J. Mol. Sci..

[23] Patel M.R., Peterson E.D., Dai D., Brennan J.M., Redberg R.F., Anderson H.V., Brindis R.G., Douglas P.S. (2010). Low diagnostic yield of elective coronary angiography. N. Engl. J. Med..

[24] Kunadian V., Chieffo A., Camici P.G., Berry C., Escaned J., Maas A.H.E.M., Prescott E., Karam N., Appelman Y., Fraccaro C. (2020). An EAPCI expert consensus document on ischaemia with non-obstructive coronary arteries in collaboration with European Society of Cardiology Working Group on Coronary Pathophysiology & Microcirculation Endorsed by Coronary Vasomotor Disorders International Study Group. Eur. Heart J..

[25] Vrints C., Andreotti F., Koskinas K.C., Rossello X., Adamo M., Ainslie J., Banning A.P., Budaj A., Buechel R.R., Chiariello G.A. (2024). 2024 ESC guidelines for the management of chronic coronary syndromes: Developed by the task force for the management of chronic coronary syndromes of the European Society of Cardiology (ESC) endorsed by the European Association for Cardio-Thoracic Surgery (EACTS). Eur. Heart J..

[26] American Diabetes Association Professional Practice Committee (2024). Diagnosis and classification of diabetes: Standards of care in diabetes—2024. Diabetes Care.

[27] McEvoy J.W., McCarthy C.P., Bruno R.M., Brouwers S., Canavan M.D., Ceconi C., Christodorescu R.M., Daskalopoulou S.S., Ferro C.J., Gerdts E. (2024). 2024 ESC Guidelines for the management of elevated blood pressure and hypertension: Developed by the task force on the management of elevated blood pressure and hypertension of the European Society of Cardiology (ESC) and endorsed by the European Society of Endocrinology (ESE) and the European Stroke Organisation (ESO). Eur. Heart J..

[28] Visseren F.L., Mach F., Smulders Y.M., Carballo D., Koskinas K.C., Bäck M., Benetos A., Biffi A., Boavida J.-M., Capodanno D. (2021). 2021 ESC Guidelines on cardiovascular disease prevention in clinical practice: Developed by the Task Force for cardiovascular disease prevention in clinical practice with representatives of the European Society of Cardiology and 12 medical societies with the special contribution of the European Association of Preventive Cardiology (EAPC). Eur. Heart J..

[29] Toldo S., Mauro A.G., Cutter Z., Abbate A. (2018). Inflammasome, pyroptosis, and cytokines in myocardial ischemia-reperfusion injury. Am. J. Physiol.-Heart Circ. Physiol..

[30] Hansson G.K. (2005). Inflammation, atherosclerosis, and coronary artery disease. N. Engl. J. Med..

[31] Tamhane U.U., Aneja S., Montgomery D., Rogers E.-K., Eagle K.A., Gurm H.S. (2008). Association between admission neutrophil to lymphocyte ratio and outcomes in patients with acute coronary syndrome. Am. J. Cardiol..

[32] González-Pacheco H., Amezcua-Guerra L.M., Sandoval J., Martínez-Sánchez C., Ortiz-León X.A., Peña-Cabral M.A., Bojalil R. (2017). Prognostic implications of serum albumin levels in patients with acute coronary syndromes. Am. J. Cardiol..

[33] Azab B., Zaher M., Weiserbs K.F., Torbey E., Lacossiere K., Gaddam S., Gobunsuy R., Jadonath S., Baldari D., McCord D. (2010). Usefulness of neutrophil to lymphocyte ratio in predicting short-and long-term mortality after non–ST-elevation myocardial infarction. Am. J. Cardiol..

[34] Park J.J., Jang H.-J., Oh I.-Y., Yoon C.-H., Suh J.-W., Cho Y.-S., Youn T.-J., Cho G.-Y., Chae I.-H., Choi D.-J. (2013). Prognostic value of neutrophil to lymphocyte ratio in patients presenting with ST-elevation myocardial infarction undergoing primary percutaneous coronary intervention. Am. J. Cardiol..

[35] Arbel Y., Finkelstein A., Halkin A., Birati E.Y., Revivo M., Zuzut M., Shevach A., Berliner S., Herz I., Keren G. (2012). Neutrophil/lymphocyte ratio is related to the severity of coronary artery disease and clinical outcome in patients undergoing angiography. Atherosclerosis.

[36] Arefnia M., Bayat M., Hosseinzadeh E., Basiri E.A., Ghodsirad M., Naghshineh R., Zamani H. (2025). The predictive value of CRP/albumin ratio (CAR) in the diagnosis of ischemia in myocardial perfusion scintigraphy. Hipertens. Riesgo Vasc..

[37] Sabanoglu C., Inanc I. (2022). C-reactive protein to albumin ratio predicts for severity of coronary artery disease and ischemia. Eur. Rev. Med. Pharmacol. Sci..

[38] Ozdemir S., Barutcu A., Gazi E., Tan Y., Turkon H. (2015). The relationship between some complete blood count parameters and myocardial perfusion: A scintigraphic approach. World J. Nucl. Med..

[39] Unkun T., Fidan S., Derebey S.T., Şengör B.G., Aytürk M., Sarı M., Efe S.Ç., Alıcı G., Özkan B., Karagöz A. (2024). The Value of Naples Prognostic Score in Predicting Ischemia on Myocardial Perfusion Scintigraphy. Koşuyolu Heart J..

[40] Efe S.Ç., Candan Ö.Ö., Gündoğan C., Öz A., Yüksel Y., Ayca B., Çermik T.F. (2020). Value of C-reactive protein/albumin ratio for predicting ischemia in myocardial perfusion scintigraphy. Mol. Imaging Radionucl. Ther..

[41] Don B.R., Kaysen G. (2004). Poor nutritional status and inflammation: Serum albumin: Relationship to inflammation and nutrition. Seminars in Dialysis.

[42] Lai G., Zhao Y., Yang C., Zheng Y., Sun J., Zhao Y., Ding M. (2025). Association of Naples prognostic score with cardiovascular disease risk and its longitudinal prognostic impact on mortality in cardiovascular disease patients: Evidence from NHANES. Nutrition, Metabolism and Cardiovascular Diseases. Nutr. Metab. Cardiovasc. Dis..

[43] Zhao Z., Zhang X., Sun T., Huang X., Ma M., Yang S., Zhou Y. (2024). Prognostic value of systemic immune-inflammation index in CAD patients: Systematic review and meta-analyses. Eur. J. Clin. Investig..

[44] Erdoğan M., Erdöl M.A., Öztürk S., Durmaz T. (2020). Systemic immune-inflammation index is a novel marker to predict functionally significant coronary artery stenosis. Biomark. Med..

[45] Ibrahim H., Schutt R.C., Hannawi B., DeLao T., Barker C.M., Kleiman N.S. (2014). Association of immature platelets with adverse cardiovascular outcomes. J. Am. Coll. Cardiol..

[46] Coppinger J.A., Cagney G., Toomey S., Kislinger T., Belton O., McRedmond J.P., Cahill D.J., Emili A., Fitzgerald D.J., Maguire P.B. (2004). Characterization of the proteins released from activated platelets leads to localization of novel platelet proteins in human atherosclerotic lesions. Blood.

[47] Pitsilos S., Hunt J., Mohler E., Prabhakar A., Poncz M., Dawicki J., Khalapyan T., Wolfe M.L., Fairman R., Mitchell M. (2003). Platelet factor 4 localization in carotid atherosclerotic plaques: Correlation with clinical parameters. Thromb. Haemost..

