# Supplementary material for: The Value of the Naples Prognostic Score and the Systemic Immune-Inflammation Index in Predicting Ischemia on Myocardial Perfusion Scintigraphy
Source: Diagnostics (Basel). 2025 May 29;15(11):1372. doi: 10.3390/diagnostics15111372 (PMC12154419; doi:10.3390/diagnostics15111372)
Supplement: Supplementary file 1 [file diagnostics-15-01372-s001.zip › diagnostics-3644303-supplementary.pdf]

Table S1. ROC analysis results of NLR, LMR and SII measurements in differentiating ischemia and non-ischemia groups

	**Definitions**	**NLR**	**LMR**	**SII**
**AUC**		0.656	0.534	0.588
**95% CI**		0.611-0.700	0.487-0.581	0.542-0.634
**p-value**		**<0.001**	0.159	**<0.001**
**The best cut-off point**		>2.04	n/a	>528.27
**Sensitivity**	TP / (TP+FN)	150 / 239 (62.8%)	n/a	116 / 239 (48.5%)
**Specificity**	TN / (TN+FP)	240 / 376 (63.8%)	n/a	258 / 376 (68.6%)
**PPV**	TP / (TP+FP)	150 / 286 (52.4%)	n/a	116 / 234 (49.5%)
**NPV**	TN / (FN+TN)	240 / 329 (72.9%)	n/a	258 / 381 (67.7%)
**Accuracy**	(TP+TN) / N	390 / 615 (63.4%)	n/a	374 / 615 (60.9%)

AUC: Area Under the Curve, CI: Confidence Interval, TP: True Positive, FN: False Negative, TN: True Negative, FP: False Positive, PPV: Positive Predictive Value, NPV: Negative Predictive Value.
